# Supplementary material for: Bilirubin Metabolism and Thyroid Cancer: Insights from ALBI and PALBI Indices
Source: Biomolecules. 2025 Jul 18;15(7):1042. doi: 10.3390/biom15071042 (PMC12293926; doi:10.3390/biom15071042)
Supplement: Supplementary file 1 [file biomolecules-15-01042-s001.zip › Supplementary Figure.pptx]

## Slide 1
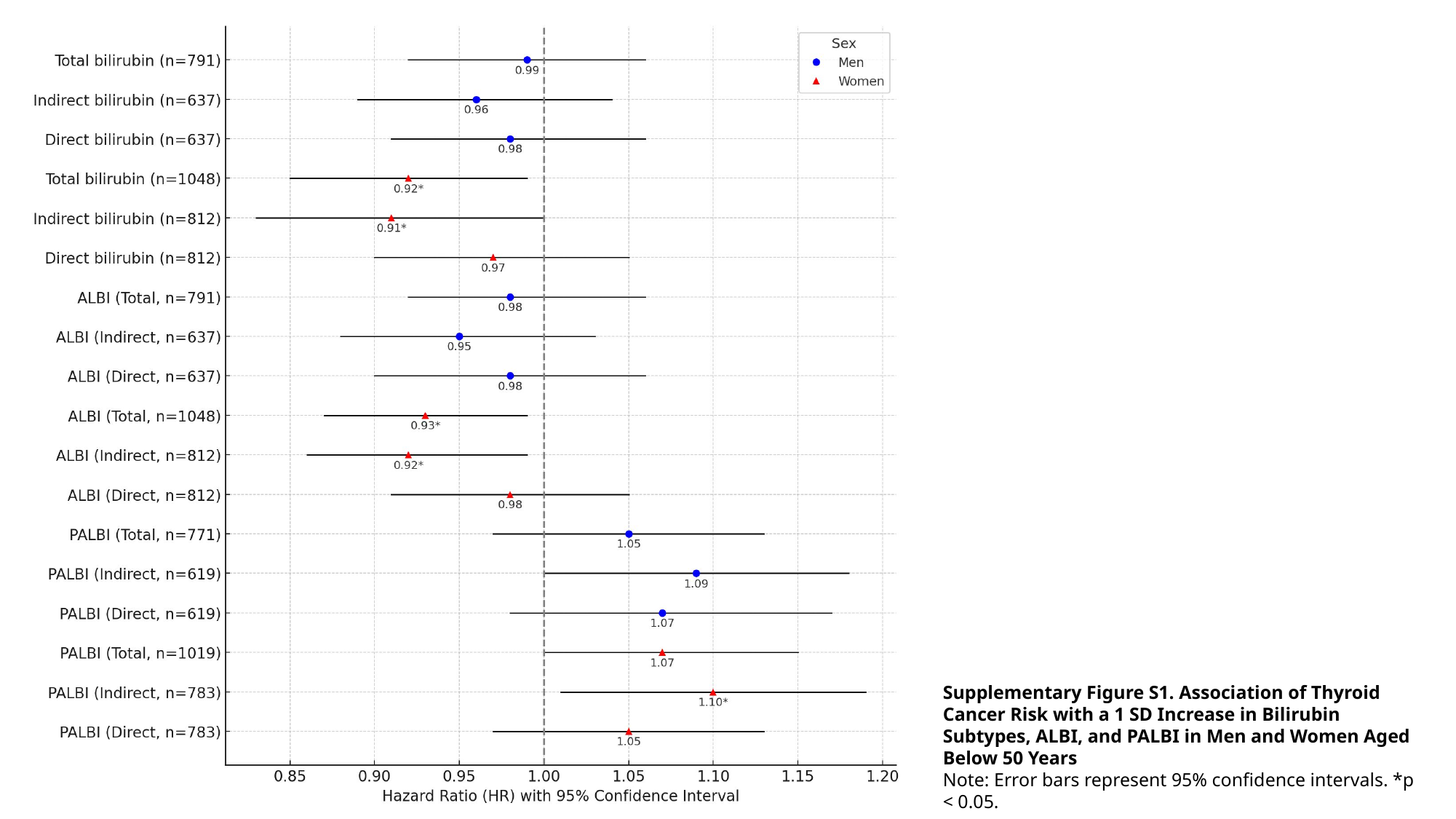

Supplementary Figure S1. Association of Thyroid Cancer Risk with a 1 SD Increase in Bilirubin Subtypes, ALBI, and PALBI in Men and Women Aged Below 50 Years
Note: Error bars represent 95% confidence intervals. *p < 0.05.
