# Supplementary material for: Bilirubin Metabolism and Thyroid Cancer: Insights from ALBI and PALBI Indices
Source: Biomolecules. 2025 Jul 18;15(7):1042. doi: 10.3390/biom15071042 (PMC12293926; doi:10.3390/biom15071042)
Supplement: Supplementary file 1 [file biomolecules-15-01042-s001.zip › Supplementary Tables.pdf]

Supplementary Table S1. Association of Thyroid Cancer Risk with a 1 SD Increase in Bilirubin Subtypes, ALBI, and PALBI According to Smoking Status in Men

Men	Bilirubin Subtypes	Status	Case	HR (95% CI) §	*p*-value
Bilirubin	Total Bilirubin	NS	186	0.91 (0.79-1.05)	0.2095
		FS	229	0.93 (0.82-1.07)	0.3130
		CS	293	1.01 (0.90-1.14)	0.8351
		ES	552	0.98 (0.90-1.07)	0.7217
	Indirect Bilirubin	NS	186	0.89 (0.77-1.04)	0.1374
		FS	229	0.91 (0.79-10.4)	0.1688
		CS	293	1.03 (0.91-1.16)	0.6723
		ES	522	0.98 (0.90-1.07)	0.6346
	Direct Bilirubin	NS	186	0.96 (0.83-1.10)	0.5326
		FS	229	0.99 (0.87-1.12)	0.8766
		CS	293	0.99 (0.88-1.11)	0.8326
		ES	552	1.00 (0.91-1.09)	0.9374
ALBI	Total Bilirubin	NS	186	0.91 (0.78-1.05)	0.1894
		FS	229	0.94 (0.82-1.07)	0.3310
		CS	293	1.01 (0.89-1.13)	0.9279
		ES	522	0.98 (0.90-1.08)	0.7063
	Indirect Bilirubin	NS	186	0.89 (0.76-1.03)	0.1164
		FS	229	0.92 (0.80-1.05)	0.1980
		CS	293	1.01 (0.90-1.14)	0.8237
		ES	522	0.98 (0.89-1.07)	0.6162
	Direct Bilirubin	NS	186	0.95 (0.81-1.11)	0.5345
		FS	229	0.99 (0.86-1.14)	0.8652
		CS	293	0.99 (0.87-1.12)	0.8427
		ES	522	1.00 (0.91-1.09)	0.9325
PALBI	Total Bilirubin	NS	186	1.08 (0.93-1.25)	0.3053
		FS	229	**1.14 (1.00-1.31)**	**0.0583**
		CS	293	1.00 (0.89-1.13)	0.9978
		ES	522	1.05 (0.96-1.15)	0.2767
	Indirect Bilirubin	NS	186	1.08 (0.94-1.25)	0.2869
		FS	229	**1.18 (1.03-1.35)**	**0.0181**
		CS	293	1.00 (0.88-1.12)	0.9341
		ES	522	1.06 (0.97-1.16)	0.1752
	Direct Bilirubin	NS	186	1.02 (0.88-1.19)	0.7733
		FS	229	1.14 (0.99-1.31)	0.0679
		CS	293	1.03 (0.91-1.16)	0.6969
		ES	522	1.07 (0.97-1.17)	0.1793

Abbreviations: NS, never smokers; FS, former smokers; CS, current smokers; ES, ever smokers (includes former and current smokers)

§The Cox proportional hazards model was adjusted for age, alcohol use body mass index, GOT, GGT, BUN, and family history.

Supplementary Table S2. Association of Thyroid Cancer Risk with a 1 SD Increase in Bilirubin Subtypes, ALBI, and PALBI According to Smoking Status in Women

Women	Bilirubin Subtypes	Status	Case	HR (95% CI) §	*p*-value
Bilirubin	Total Bilirubin	NS	782	0.93 (0.85-1.02)	0.1236
		FS	81	0.89 (0.65-1.20)	0.4323
		CS	38	1.19 (0.83-1.71)	0.3340
		ES	119	0.99 (0.78-1.24)	0.9098
	Indirect Bilirubin	NS	782	**0.91 (0.83-1.00)**	**0.0534**
		FS	81	0.85 (0.61-1.17)	0.3185
		CS	38	1.17 (0.80-1.72)	0.4145
		ES	119	0.95 (0.74-1.22)	0.6928
	Direct Bilirubin	NS	782	0.98 (0.90-1.06)	0.5707
		FS	81	0.97 (0.77-1.22)	0.7812
		CS	38	1.18 (0.86-1.62)	0.3082
		ES	119	1.03 (0.85-1.24)	0.7607
ALBI	Total Bilirubin	NS	782	0.94 (0.88-1.02)	0.1254
		FS	81	0.88 (0.70-1.12)	0.3040
		CS	38	1.14 (0.83-1.57)	0.4175
		ES	119	0.96 (0.80-1.16)	0.6885
	Indirect Bilirubin	NS	782	**0.93 (0.86-1.00)**	**0.0596**
		FS	81	0.83 (0.68-1.02)	0.0810
		CS	38	1.13 (0.81-1.57)	0.4849
		ES	119	0.90 (0.75-1.08)	0.2638
	Direct Bilirubin	NS	782	0.99 (0.92-1.06)	0.6823
		FS	81	1.02 (0.82-1.26)	0.8857
		CS	38	1.15 (0.83-1.57)	0.4025
		ES	119	1.05 (0.88-1.26)	0.5653
PALBI	Total Bilirubin	NS	782	**1.12 (1.03-1.22)**	**0.0081**
		FS	81	0.95 (0.73-1.22)	0.6586
		CS	38	0.87 (0.61-1.24)	0.4325
		ES	119	0.92 (0.75-1.13)	0.4343
	Indirect Bilirubin	NS	782	**1.14 (1.05-1.23)**	**0.0023**
		FS	81	0.92 (0.72-1.17)	0.5010
		CS	38	0.90 (0.63-1.29)	0.5637
		ES	119	0.92 (0.75-1.12)	0.3969
	Direct Bilirubin	NS	782	**1.10 (1.02-1.19)**	**0.0156**
		FS	81	0.88 (0.73-1.08)	0.2232
		CS	38	0.91 (0.65-1.28)	0.5768
		ES	119	0.89 (0.75-1.06)	0.1775

Abbreviations: NS, never smokers; FS, former smokers; CS, current smokers; ES, ever smokers (includes former and current smokers)

§The Cox proportional hazards model was adjusted for age, alcohol use body mass index, GOT, GGT, BUN, and family history.

Supplementary Table S3. Association of Thyroid Cancer Risk with a 1 SD Increase in Bilirubin Subtypes, ALBI, and PALBI According to Alcohol Drinking Status in Men

Men	Bilirubin Subtypes	Status	Case	HR (95% CI) §	*p*-value
Bilirubin	Total Bilirubin	ND	33	0.92 (0.62-1.36)	0.6676
		FD	58	0.83 (0.62-1.10)	0.1902
		CD	617	0.97 (0.90-1.06)	0.5139
		ED	675	0.96 (0.89-1.04)	0.3230
	Indirect Bilirubin	ND	33	0.87 (0.58-1.31)	0.5146
		FD	58	0.79 (0.59-1.06)	0.1153
		CD	617	0.97 (0.89-1.05)	0.4467
		ED	675	0.95 (0.88-1.03)	0.2388
	Direct Bilirubin	ND	33	1.01 (0.70-1.46)	0.9571
		FD	58	0.92 (0.70-1.21)	0.5393
		CD	617	0.99 (0.91-1.07)	0.7322
		ED	675	0.98 (0.91-1.06)	0.6385
ALBI	Total Bilirubin	ND	33	0.95 (0.66-1.37)	0.7864
		FD	58	0.86 (0.66-1.11)	0.2378
		CD	617	0.97 (0.89-1.05)	0.4601
		ED	675	0.96 (0.89-1.04)	0.2971
	Indirect Bilirubin	ND	33	0.95 (0.67-1.35)	0.7806
		FD	58	0.82 (0.63-1.06)	0.1246
		CD	617	0.96 (0.89-1.05)	0.3847
		ED	675	0.95 (0.88-1.03)	0.2007
	Direct Bilirubin	ND	33	0.99 (0.68-1.45)	0.9640
		FD	58	0.94 (0.72-1.23)	0.6725
		CD	617	0.98 (0.90-1.07)	0.7111
		ED	675	0.98 (0.90-1.07)	0.6513
PALBI	Total Bilirubin	ND	33	1.30 (0.88-1.92)	0.1894
		FD	58	1.27 (0.96-1.69)	0.0951
		CD	617	1.04 (0.96-1.13)	0.3929
		ED	675	1.05 (0.97-1.14)	0.1961
	Indirect Bilirubin	ND	33	1.38 (0.94-2.01)	0.0983
		FD	58	1.31 (0.99-1.73)	0.0585
		CD	617	1.04 (0.96-1.13)	0.3404
		ED	675	1.06 (0.98-1.15)	0.1436
	Direct Bilirubin	ND	33	1.31 (0.91-1.89)	0.1411
		FD	58	1.20 (0.91-1.59)	0.1946
		CD	617	1.03 (0.95-1.12)	0.4839
		ED	675	1.04 (0.96-1.13)	0.3014

Abbreviations: CI, confidence interval; HR, hazard ratio; ND, never drinkers; FD, former drinkers; CD, current drinkers; ED, ever drinkers (includes former and current drinkers).

§The Cox proportional hazards model was adjusted for age, smoking status, body mass index, GOT, GGT, BUN, and family history.

Supplementary Table S4. Association of Thyroid Cancer Risk with a 1 SD Increase in Bilirubin Subtypes, ALBI, and PALBI According to Alcohol Drinking Status in Women

Women	Bilirubin Subtypes	Status	Case	HR (95% CI) §	*p*-value
Bilirubin	Total Bilirubin	ND	277	0.93 (0.80-1.09)	0.3982
		FD	190	0.86 (0.71-1.05)	0.1329
		CD	434	0.97 (0.86-1.09)	0.5583
		ED	624	0.93 (0.84-1.03)	0.1680
	Indirect Bilirubin	ND	277	0.90 (0.77-1.06)	0.2217
		FD	190	0.84 (0.68-1.02)	0.0842
		CD	434	0.96 (0.85-1.08)	0.4534
		ED	624	0.92 (0.83-1.02)	0.1094
	Direct Bilirubin	ND	277	1.00 (0.87-1.15)	0.9728
		FD	190	0.93 (0.78-1.11)	0.4198
		CD	434	0.99 (0.89-1.10)	0.8303
		ED	624	0.97 (0.88-1.06)	0.4615
ALBI	Total Bilirubin	ND	277	0.93 (0.82-1.06)	0.2851
		FD	190	0.91 (0.78-1.05)	0.2030
		CD	434	0.97 (0.88-1.07)	0.5064
		ED	624	0.95 (0.87-1.03)	0.1805
	Indirect Bilirubin	ND	277	0.91 (0.80-1.03)	0.1258
		FD	190	0.89 (0.76-1.03)	0.1234
		CD	434	0.96 (0.87-1.06)	0.3714
		ED	624	0.93 (0.86-1.02)	0.1061
	Direct Bilirubin	ND	277	1.01 (0.89-1.14)	0.9290
		FD	190	0.97 (0.84-1.11)	0.6385
		CD	434	0.99 (0.90-1.09)	0.8824
		ED	624	0.98 (0.91-1.06)	0.5951
PALBI	Total Bilirubin	ND	277	1.13 (0.97-1.30)	0.1144
		FD	190	1.18 (0.99-1.40)	0.0636
		CD	434	1.05 (0.94-1.17)	0.3922
		ED	624	1.09 (0.99-1.20)	0.0709
	Indirect Bilirubin	ND	277	**1.15 (1.00-1.32)**	**0.0543**
		FD	190	**1.18 (1.00-1.39)**	**0.0488**
		CD	434	1.06 (0.95-1.17)	0.3253
		ED	624	**1.09 (1.00-1.20)**	**0.0506**
	Direct Bilirubin	ND	277	1.09 (0.96-1.24)	0.1738
		FD	190	1.12 (0.96-1.30)	0.1584
		CD	434	1.04 (0.94-1.15)	0.4828
		ED	624	1.06 (0.98-1.16)	0.1470

Abbreviations: CI, confidence interval; HR, hazard ratio; ND, never drinkers; FD, former drinkers; CD, current drinkers; ED, ever drinkers (includes former and current drinkers).

§The Cox proportional hazards model was adjusted for age, smoking status, body mass index, GOT, GGT, BUN, and family history.

Supplementary Table S5. Sensitivity Analysis: Association of Thyroid Cancer Risk with a 1 SD Increase in Bilirubin Subtypes, ALBI, and PALBI in Men and Women of All Ages

	Bilirubin Subtypes	Case	HR (95% CI) §	*p*-value
**Men**	Total Bilirubin	966	1.00 (0.94-1.06)	0.8922
	Indirect Bilirubin	748	0.97 (0.91-1.04)	0.4139
	Direct Bilirubin	748	0.98 (0.91-1.06)	0.6174
**Women**	Total Bilirubin	1348	0.95 (0.89-1.02)	0.1356
	Indirect Bilirubin	960	**0.92 (0.85-1.00)**	**0.0442**
	Direct Bilirubin	960	0.99 (0.92-1.07)	0.8250
	**ALBI Index**			
**Men**	Total Bilirubin	966	1.00 (0.94-1.07)	0.9600
	Indirect Bilirubin	748	0.97 (0.90-1.04)	0.3758
	Direct Bilirubin	748	0.98 (0.91-1.06)	0.6279
**Women**	Total Bilirubin	1348	0.96 (0.91-1.01)	0.1410
	Indirect Bilirubin	960	**0.93 (0.87-1.00)**	**0.0336**
	Direct Bilirubin	960	1.00 (0.94-1.06)	0.9604
	**PALBI Index**			
**Men**	Total Bilirubin	935	1.03 (0.97-1.10)	0.3365
	Indirect Bilirubin	719	1.07 (0.99-1.15)	0.1038
	Direct Bilirubin	719	1.05 (0.97-1.13)	0.2478
**Women**	Total Bilirubin	1307	1.06 (0.99-1.13)	0.0964
	Indirect Bilirubin	919	**1.09 (1.01-1.18)**	**0.0199**
	Direct Bilirubin	919	1.05 (0.98-1.12)	0.1850

Abbreviations: CI, confidence interval; HR, hazard ratio.

§The Cox proportional hazards model was adjusted for age, smoking status, alcohol use, body mass index, and family history.
